# Excessive external rotation of the ipsilateral iliac wing following Pemberton acetabuloplasty: a three-dimensional computed tomography study

**DOI:** 10.1186/s13018-026-06790-8

**Published:** 2026-03-27

**Authors:** Yu-Han Chen, Kuan-Wen Wu, Chih-Kai Hong, Chia-Che Lee, Yu-Hung Chen, Ken N. Kuo, Ting-Ming Wang

**Affiliations:** 1 Department of Orthopaedic Surgery, National Taiwan University Hospital, No.7, Zhongshan S. Rd., Zhongzheng District, Taipei City, 10002 Taiwan; 2 Graduate Institute of Biomedical Electronics and Bioinformatics, National Taiwan University, No. 1, Sec. 4, Roosevelt Rd., Da’an Dist, Taipei City, 106319 Taiwan; 3 Cochrane Taiwan, Taipei Medical University, No. 250 Wuxing Street, Taipe City, 11031 Taiwan

**Keywords:** Developmental dysplasia of the hip, Pemberton acetabuloplasty, Pelvic rotational alignment, Innominate bone morphology, Acetabular anteversion, Three-dimensional computed tomography, Abnormal gait mechanics

## Abstract

**Background:**

Emerging evidence suggests that developmental dysplasia of the hip involves not only localized acetabular dysplasia but also internal rotation of the entire innominate bone. Pemberton acetabuloplasty is widely used to improve acetabular coverage in walking-age children and generally yields favorable radiographic and clinical outcomes. However, the effect of this procedure on the global rotational alignment of the innominate bone has not been systematically investigated.

**Methods:**

This retrospective comparative study included patients with a primary diagnosis of developmental dysplasia of the hip who underwent Pemberton acetabuloplasty at a tertiary care center between January 2011 and October 2021. Postoperative pelvic computed tomography scans were available for 20 unilateral and 12 bilateral cases, all of which were included in the analysis. In unilateral cases, the operated hemipelvis was compared with the contralateral side using paired t-tests to minimize inter-individual variability. Pelvic rotational parameters were further compared between unilateral and bilateral groups. Finally, data from all operated hips were pooled to evaluate the association between postoperative innominate rotational alignment and acetabular anteversion using Pearson’s correlation coefficient.

**Results:**

In unilateral cases, the operated side demonstrated significantly greater external rotation of the upper pelvis compared with the contralateral side (mean difference, 3.3°; 95% confidence interval [CI], 0.7°–5.9°; *p* = 0.002), whereas acetabular anteversion and lower pelvic rotation did not differ significantly. No significant differences in pelvic rotational parameters or acetabular anteversion were observed between unilateral and bilateral procedures. When all operated hips were pooled for analysis, lower pelvic rotation demonstrated a moderate positive correlation with acetabular anteversion (*r* = 0.614, *p* < 0.001), while upper pelvic rotation showed a weak but statistically significant correlation (*r* = 0.308, *p* = 0.042).

**Conclusions:**

Postoperative excessive external rotation of the upper hemipelvis was observed following Pemberton acetabuloplasty, suggesting that structural alterations may extend beyond localized acetabular reshaping. These findings offer additional insight into the broader morphological changes associated with the procedure. Further prospective studies incorporating longitudinal imaging and objective functional assessment are warranted to better define the clinical significance of these structural alterations.

***Level of Evidence*:**

Level III, Retrospective comparative study.

## Background

 Hip dysplasia and femoroacetabular impingement are recognized as two principal structural precursors of hip osteoarthritis [1], with developmental dysplasia of the hip (DDH) identified in approximately 20–40% of affected individuals [2]. DDH is characterized by insufficient acetabular coverage of the femoral head [3], resulting in abnormal stress distribution and increased joint contact pressure on the articular cartilage, which ultimately predispose patients to early-onset secondary hip osteoarthritis [4–7]. Although DDH has traditionally been regarded as a localized acetabular disorder, accumulating evidence suggests that it is associated with pathomorphologic changes involving the entire pelvis [8, 9]. In particular, several studies have reported internal rotational of the entire innominate bone, extending from the iliac wing to the ischiopubic region, in patients with DDH [8–11].

Treatment strategies for DDH are largely age-dependent, reflecting the diminishing remodeling capacity of the acetabulum over time. Whereas concentric reduction during infancy permits adequate acetabular remodeling, children who present after walking age frequently require pelvic osteotomy to restore hip stability and improve femoral head coverage [12]. A 2016 systematic review of DDH management in walking-age children concluded that open reduction with concomitant pelvic osteotomy is the most appropriate option to provide durable results with the lowest risk of avascular necrosis and best radiological and clinical results [13].

Among the various pelvic osteotomies, Pemberton acetabuloplasty is widely utilized for the treatment of DDH in walking-age children. The procedure reshapes the acetabular roof in a caudal and anterior direction, using the triradiate cartilage as a hinge following an incomplete iliac osteotomy [14]. Long-term follow-up studies extending beyond 10 years have demonstrated favorable functional outcomes, as assessed by the SF-36 and Harris Hip Score [15]. Nevertheless, residual gait abnormalities and increased hip joint loading have been reported despite surgical intervention [16, 17]. Gait analyses of adolescents who underwent unilateral Pemberton acetabuloplasty in early childhood have shown persistent elevations in joint loading on both the operated and contralateral limbs compared with healthy controls, findings that have been discussed in relation to the potential risk of premature osteoarthritis [16]. These patients have also been reported to exhibit altered pelvic kinematics, including increased anterior pelvic tilt in the sagittal plane, pelvic hiking on the operated side in the coronal plane, and pelvic rotation toward the contralateral side in the axial plane, accompanied by compensatory changes at the knee and ankle [17].

Although postoperative acetabular morphology following Pemberton acetabuloplasty has been well described [15, 18], its potential effect on the rotational alignment of the entire pelvis has not been systematically evaluated. We hypothesized that Pemberton acetabuloplasty may be associated with measurable postoperative rotational asymmetry of the innominate bone beyond localized acetabular reshaping. Accordingly, the present study aimed to assess postoperative changes in the rotational alignment of the entire innominate bone using three-dimensional computed tomography.

## Methods

### Study design and setting

This retrospective comparative study was approved by the institutional ethics committee and conducted in accordance with the Declaration of Helsinki. We reviewed the medical records and imaging studies of patients with a primary diagnosis of developmental dysplasia of the hip (DDH) who underwent Pemberton acetabuloplasty at our tertiary care center between January 2011 and October 2021. Since pelvic computed tomography (CT) is not routinely performed following DDH treatment, only patients with available postoperative pelvic CT scans were included in the analysis. Formal gait analysis was not performed as part of this study and is not part of the treatment protocol for these patients at our institution.

To minimize inter-individual variability, the effect of Pemberton acetabuloplasty on hemipelvic rotational alignment was evaluated in the unilateral DDH group by comparing the operated hemipelvis with the contralateral side using paired t-tests. Pelvic rotational parameters were further compared between unilateral and bilateral groups to assess whether bilateral procedures produced different rotational effects. Finally, data from both groups were pooled to examine the association between postoperative ipsilateral innominate rotation and acetabular anteversion using Pearson’s correlation coefficient.

### Description of surgery

At our institution, Pemberton acetabuloplasty is routinely performed through an anterior iliofemoral approach. When open reduction is indicated, the rectus femoris tendon is detached to facilitate surgical exposure and subsequently reattached at the conclusion of the procedure. The iliopsoas tendon is routinely released, and intra-articular obstructing structures, including the ligamentum teres, pulvinar tissue, and transverse acetabular ligament, are removed. Femoral head stability is then assessed in the neutral position as well as in abduction and internal rotation. A curved osteotomy is created along both the inner and outer cortices of the ilium and extended toward the posterior limb of the triradiate cartilage. The acetabular fragment is then rotated caudally and anteriorly, hinging on the triradiate cartilage, to enhance anterolateral coverage of the femoral head. A triangular bone graft harvested from the iliac crest proximal to the anterior superior iliac spine is inserted into the osteotomy site [14]. Postoperatively, the patient is immobilized in a hip spica cast for six weeks.

### Variables, outcome measures, data sources

To minimize positioning-related measurement errors inherent to plain radiographs, computed tomography (CT) has been proposed for the assessment of acetabular anteversion. Furthermore, three-dimensional CT (3D-CT) has demonstrated superior intra- and inter-observer reliability compared with two-dimensional CT (2D-CT) [19]. Accordingly, 3D-CT is valuable for accurately characterizing the pathomorphology of DDH and for assisting surgical planning, particularly when different types of pelvic osteotomies are considered. Pelvic CT scans for the present study were obtained at our institution using multi-detector CT scanners with 16-, 32-, or 64-detector rows from various manufacturers, including Canon Medical Systems (Aquilion ONE, Otawara, Tochigi, Japan), GE Medical Systems (Revolution CT, Waukesha, WI, USA), and Siemens Healthineers (Sensation 64 and SOMATOM Definition AS+, Erlangen, Germany). Standard pelvic CT was performed with patients in the supine position, covering the region from the anterior superior iliac spines to the inferior margin of the pelvis. Three-dimensional reconstructions were subsequently generated on the respective CT workstation platforms.

**Fig. 1:**
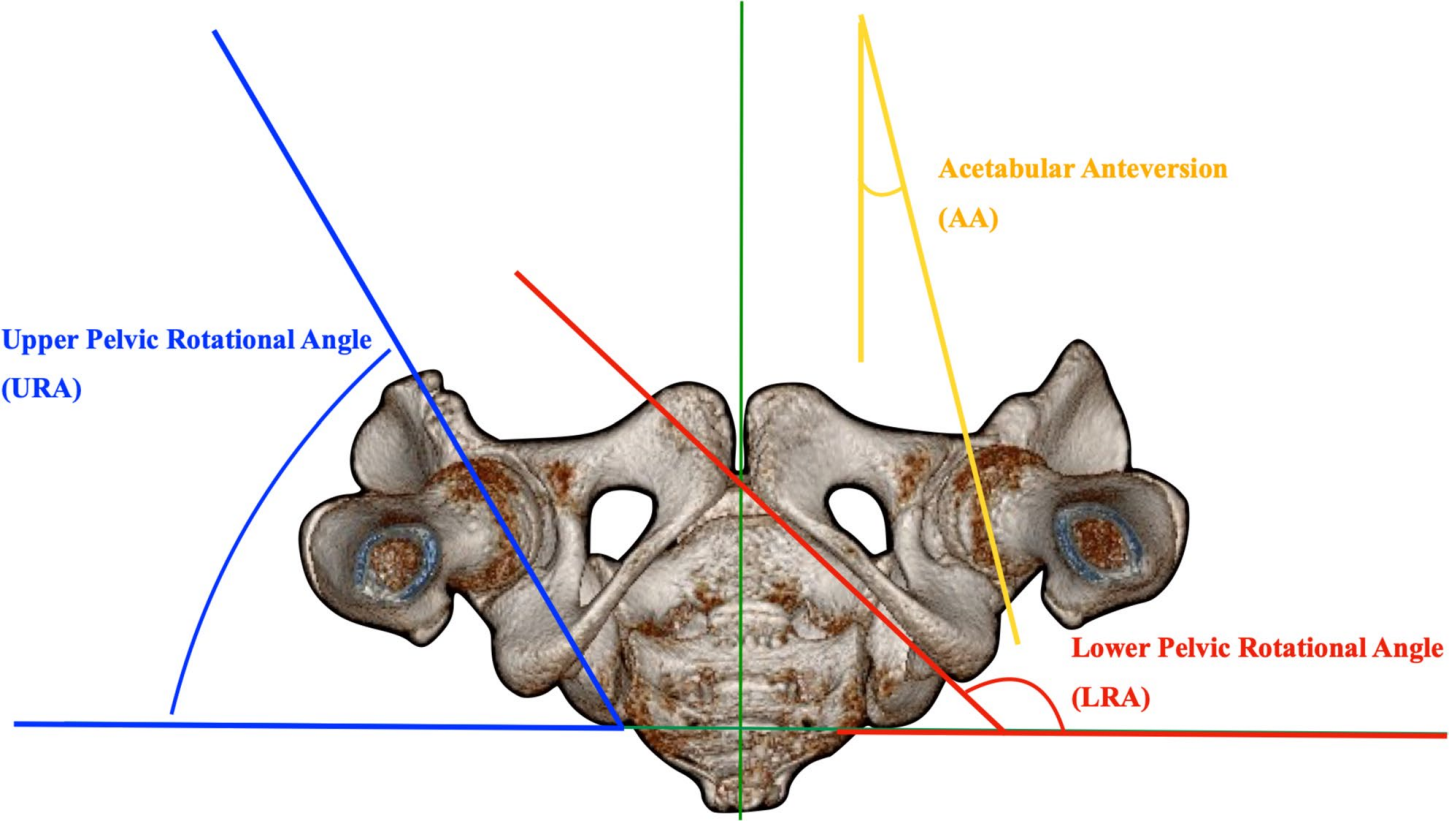
Three-dimensional pelvic computed tomography of an 11-year-old girl, obtained 5 years after right Pemberton acetabuloplasty. Acetabular anteversion (AA) was defined as the angle between a line connecting the anterior and posterior acetabular margins and the sagittal reference line bisecting the sacrum. The upper rotational angle (URA) was defined as the angle between a line connecting the midpoints of the anterior and posterior superior iliac spines and the horizontal reference line. The lower rotational angle (LRA) was defined as the angle between a line tangential to the innermost borders of the pubis and ischium and the horizontal reference line, according to the method described and validated by Jia et al. [20].

To evaluate postoperative rotational alignment of the entire innominate bone, acetabular anteversion (AA), upper rotational angle (URA), and lower rotational angle (LRA) were measured using three-dimensional computed tomography (3D CT) according to the method described and validated by Jia et al., which demonstrated excellent intra- and inter-observer reliability [20]. Three-dimensional reconstructed pelvic images were first standardized to a consistent spatial orientation. The pelvis was rotated such that the line connecting the bilateral posterior superior iliac spines (PSIS) was aligned horizontally. The reconstructed image was then adjusted in the sagittal plane to align the more caudally positioned anterior superior iliac spine (ASIS) with the anterior border of the pubic symphysis, thereby minimizing tilt-related distortion. On the caudal view of the standardized 3D reconstruction, AA was defined as the angle between a line connecting the anterior and posterior acetabular margins and the sagittal reference line bisecting the sacrum. URA was defined as the angle between a line connecting the midpoints of the anterior and posterior superior iliac spines (ASIS and PSIS) and the horizontal reference line. LRA was defined as the angle between a line tangential to the innermost borders of the pubis and ischium and the horizontal reference line (Fig. 1) [20].

### Statistical analysis

The normality of continuous variables was assessed using the Shapiro–Wilk test in conjunction with visual inspection of quantile–quantile plots. Data that satisfied normality assumptions are presented as mean with standard deviation (SD), whereas non-normally distributed data are reported as median with interquartile range (IQR). Measurement reliability was evaluated using intraclass correlation coefficients (ICC) derived from a two-way random-effects model with single measurements and absolute agreement. The upper rotational angle (URA), lower rotational angle (LRA), and acetabular anteversion (AA) satisfied normality criteria and were analyzed as normally distributed continuous variables. In patients with unilateral DDH, paired t-tests were used to compare URA, LRA, and AA between the operated and contralateral sides. Pelvic rotational parameters were further compared between unilateral and bilateral groups using two-sample t-tests. Finally, all hips were pooled to evaluate the association between postoperative innominate rotational alignment and acetabular anteversion using Pearson’s correlation coefficient. Statistical analyses were performed using Python (version 3.8) with the SciPy package (version 1.12.0).

## Results

### Participants

Between January 2011 and October 2021, 82 patients underwent Pemberton acetabuloplasty at our tertiary care center. Of these, 32 patients with available postoperative pelvic computed tomography (CT) scans were included in the analysis, comprising 20 unilateral and 12 bilateral cases. The unilateral group consisted of 4 male and 16 female patients, with left hip involvement in 13 cases and right hip involvement in 7 cases. The median age at the time of surgery was 2.5 years. Among the 20 affected hips in the unilateral group, 11 were classified as Tönnis grade I, 4 as grade II, and 1 as grade III; Tönnis grade data were unavailable for 4 hips. In the bilateral group, all 12 patients were female, with a median age at surgery of 2.0 years. Of the 24 affected hips, 8 were classified as Tönnis grade I, 6 as grade II, and 6 as grade IV, while Tönnis grade documentation was unavailable for 4 hips (Table 1).

The mean interval between surgery and postoperative CT acquisition was 88.3 months (standard deviation [SD], 66.7 months) in the unilateral group and 109.3 months (SD, 88.5 months) in the bilateral group. Baseline characteristics—including sex, age at operation, body mass index (BMI), and Tönnis grade—did not differ significantly between groups. One patient in the unilateral group was lost to follow-up at 5 months postoperatively. The remaining 31 patients, representing 43 hips, were followed for a minimum of 4 years. During the 4-year postoperative period, revision surgery was required in 2 of 43 hips. One patient underwent revision Pemberton acetabuloplasty at 13 months postoperatively due to redislocation, and another underwent Bernese periacetabular osteotomy at 30 months postoperatively for inadequate acetabular coverage.

**Table 1:** Patient characteristics

Characteristic	Unilateral DDH	Bilateral DDH	*P* value
Surgical Intervention	Unilateral Pemberton Acetabuloplasty	Bilateral Pemberton Acetabuloplasty	
Number of patients	20	12	
Median Age at Operation (yr)	2.5 (2.0-5.8)	2.0 (1.0-6.5)	0.983^a^
Sex			0.271^b^
Male	4	0	
Female	16	12	
Laterality			0.487^c^
Left	13	12	
Right	7	12	
BMI (kg/m2)	17.1 ± 3.3	18.2 ± 3.2	0.379^d^
Time from surgery to postoperative CT (months)	88.3 ± 66.7	109.3 ± 88.5	0.464^d^
Tönnis Classification for DDH (Number of Hips)			0.932^a^
Grade I	11	8	
Grade II	4	6	
Grade III	0	0	
Grade IV	1	6	
No documentation	4	4	

The data are presented as median (Interquantile range) or mean ± standard deviation. ^a^Mann-Whitney U test. ^b^Fisher’s exact test. ^c^Chi-squared test with Yates correction. ^d^ Two-sample t-test

### Reliability of measurements

All measurements obtained from three-dimensional computed tomography (3D CT) images were performed by a fifth-year orthopedic resident and repeated in a blinded manner after a one-month interval to assess intra-observer reliability. For assessment of inter-observer reliability, the same measurements of pelvic rotational parameters were independently performed by a fourth-year orthopedic resident in the same patient cohort. Agreement between measurements was evaluated using intraclass correlation coefficients (ICC) derived from a two-way random-effects model with single measurements and absolute agreement. Based on the 95% confidence intervals of the ICC estimates, values < 0.50 were interpreted as poor reliability, 0.50–0.75 as moderate reliability, 0.75–0.90 as good reliability, and > 0.90 as excellent reliability [21]. Inter-observer reliability was moderate to good for acetabular anteversion (AA) and the upper rotational angle (URA), and excellent for the lower rotational angle (LRA). Intra-observer reliability was good to excellent for AA and URA and excellent for LRA (Table 2).

**Table 2:** Intra-rater and inter-rater reliability for the three pelvic rotational angles

Intraclass correlation coefficient (95% CI)			
Reliability	URA	LRA	AA
Inter-rater reliability	0.71 (0.49, 0.84)	0.95 (0.92, 0.98)	0.75 (0.58, 0.86)
Intra-rater reliability	0.93 (0.87, 0.96)	0.96 (0.93, 0.98)	0.89 (0.81, 0.94)

URA, upper pelvic rotational angle; LRA, lower pelvic rotational angle; AA, acetabular anteversion

Using the triradiate cartilage as the anatomical boundary, the pelvis was divided into two segments: the ilium was defined as the upper segment, and the pubis and ischium as the lower segment. Following unilateral Pemberton acetabuloplasty, excessive external rotation of the upper pelvis (iliac wing) was observed on the operated side. The upper pelvic rotational angle (URA) averaged 57.9 ± 3.2° on the operated side compared with 61.2 ± 4.6° on the contralateral unaffected side (*p* = 0.002), corresponding to a mean difference of 3.3° of excessive external rotation (95% CI: 0.7°–5.9°). In contrast, there were no significant differences in acetabular anteversion (*p* = 0.675) or the lower pelvic rotational angle (*p* = 0.099) between two sides (Table 3). Overall, unilateral Pemberton acetabuloplasty, which utilizes the triradiate cartilage as a hinge, was associated with greater external rotation of the upper hemipelvis compared with the contralateral side, whereas no significant differences were observed in acetabular anteversion or lower pelvic rotational angle between sides.

**Table 3:** Comparison of pelvic rotational angles between operated and contralateral sides following unilateral Pemberton acetabuloplasty

Parameter	Operated side	Contralateral side	*P* value	Mean difference (95% CI)
URA (deg)	57.9 ± 3.2	61.2 ± 4.6	0.002^a^	− 3.3 (− 5.9, − 0.7)
LRA (deg)	129.2 ± 6.9	131.1 ± 5.4	0.099^a^	− 1.9 (− 5.9, 2.2)
AA (deg)	15.6 ± 5.8	16.0 ± 5.1	0.675^a^	− 0.4 (− 4.0, 3.2)

The data are presented as mean ± standard deviation. ^a^Paired t-test

URA, upper pelvic rotational angle; LRA, lower pelvic rotational angle; AA, acetabular anteversion

When comparing unilateral and bilateral Pemberton acetabuloplasty, no significant differences in pelvic rotational alignment were identified (Table 4). The mean upper pelvic rotational angle (URA) was 57.9° ± 3.2° in the unilateral group and 58.5° ± 4.8° in the bilateral group (*p* = 0.669). The mean lower pelvic rotational angle (LRA) was 129.2° ± 6.9° and 130.6° ± 5.8°, respectively (*p* = 0.482). These findings suggest that pelvic rotational alignment was comparable between unilateral and bilateral procedures.

**Table 4:** Pelvic rotational angles after Pemberton acetabuloplasty: comparison between unilateral and bilateral procedures

Parameter	Unilateral Pemberton	Bilateral Pemberton	*P* value	Mean difference (95% CI)
URA (deg)	57.9 ± 3.2	58.5 ± 4.8	0.669^a^	− 0.6 (− 3.1, 2.0)
LRA (deg)	129.2 ± 6.9	130.6 ± 5.8	0.482^a^	− 1.4 (− 5.4, 2.6)
AA (deg)	15.6 ± 5.8	15.4 ± 6.3	0.894^a^	0.3 (− 3.5, 4.1)

The data are presented as mean ± standard deviation. ^a^Two-sample t-test

URA, upper pelvic rotational angle; LRA, lower pelvic rotational angle; AA, acetabular anteversion

**Fig. 2:**
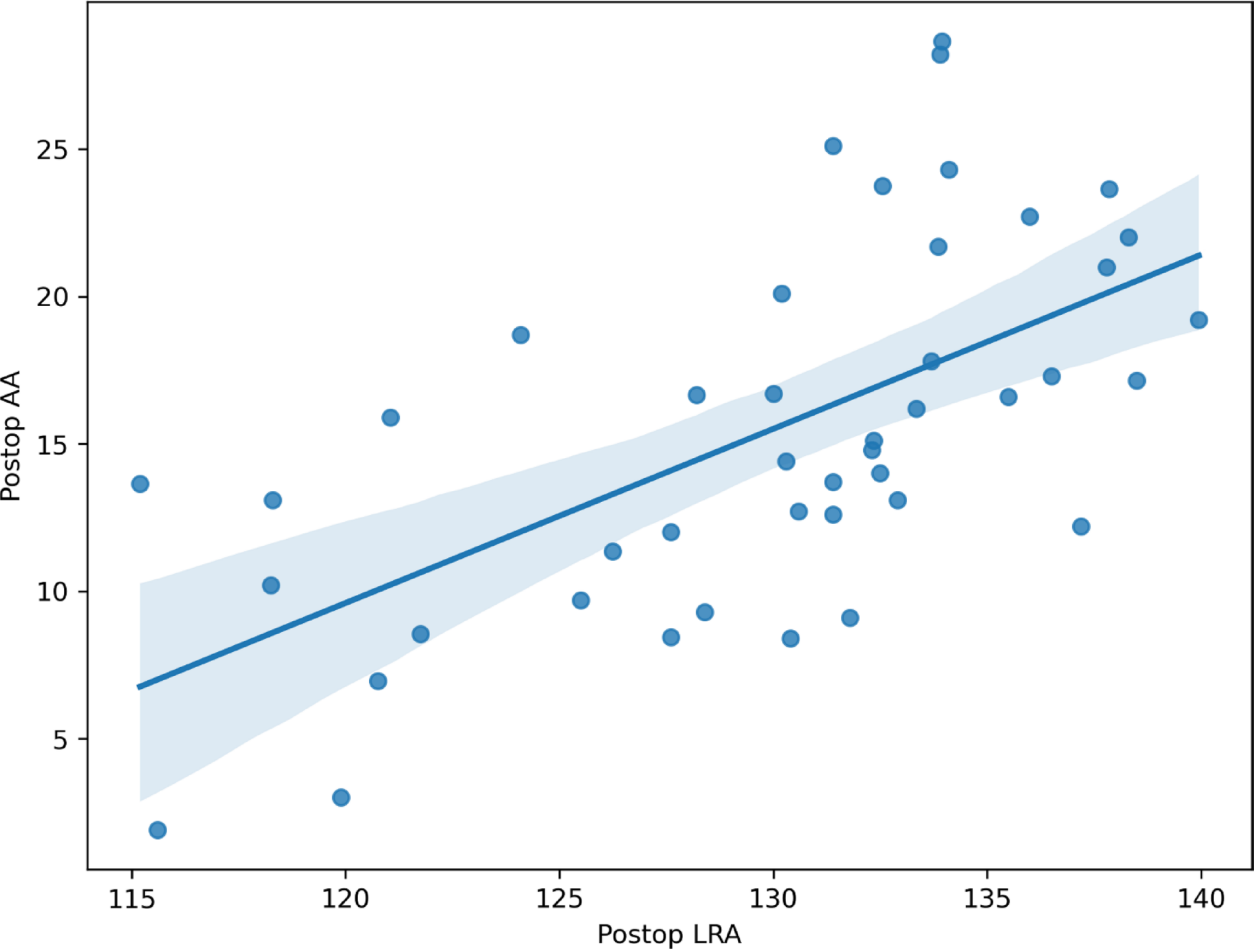
Scatter plot illustrating the moderate correlation between the lower pelvic rotational angle (LRA) and acetabular anteversion (AA). Pearson correlation coefficient *r* = 0.614 (95% CI, 0.388–0.770)

**Fig. 3:**
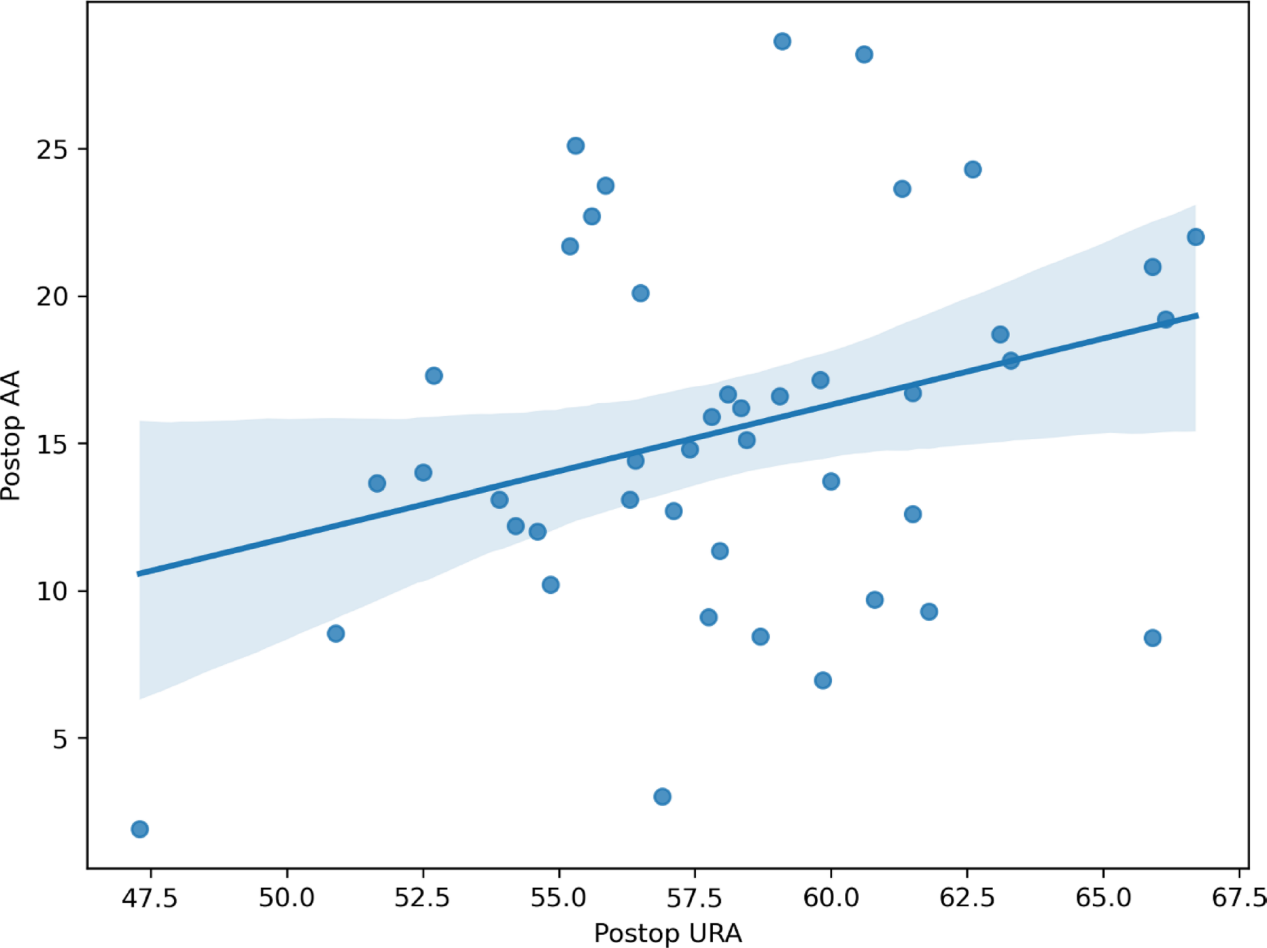
Scatter plot illustrating the weak correlation between the upper pelvic rotational angle (URA) and acetabular anteversion (AA). Pearson correlation coefficient *r* = 0.308 (95% CI, 0.013–0.554)

In the postoperative innominate bone, a moderate positive correlation was observed between the lower pelvic rotational angle (LRA) and acetabular anteversion (AA), with a Pearson correlation coefficient of 0.614 (95% confidence interval [CI], 0.388–0.770) (Table 5; Fig. 2). A statistically significant but weak correlation was also identified between the upper pelvic rotational angle (URA) and acetabular anteversion (*r* = 0.308; 95% CI, 0.013–0.554; *p* = 0.042) (Fig. 3). Given that lower LRA values correspond to greater external rotation of the lower pelvis, this positive correlation indicates that greater external rotation of the lower pelvis was associated with reduced acetabular anteversion on three-dimensional analysis. In the present cohort, the mean postoperative acetabular anteversion was 15.6° ± 5.8° in the unilateral group and 15.4° ± 6.3° in the bilateral group. No cases of acetabular retroversion were identified among the 44 hips treated with Pemberton acetabuloplasty

**Table 5:** Correlation between upper and lower pelvic rotational angles and acetabular anteversion in hips treated with Pemberton acetabuloplasty

Parameter	Acetabular ateversion (AA)	
	Pearson correlation coefficient (95% CI)	*P* value
Upper pelvic rotational angle (URA)	0.308 (0.013, 0.554) ^a^	0.042
Lower pelvic rotational angle (LRA)	0.614 (0.388, 0.770) ^a^	< 0.001

^a^ The 95% confidence interval for Pearson’s r was derived using Fisher’s z transformation

## Discussion

Although postoperative acetabular morphological changes following Pemberton acetabuloplasty have been well described [15, 18], the potential influence of this procedure on the morphology of the entire pelvis has not been systematically investigated. Previous gait studies of adolescents treated with unilateral Pemberton acetabuloplasty in early childhood have reported altered pelvic kinematics and increased joint loading [16, 17]. However, the structural correlates underlying these observations remain incompletely understood. In the present study, we focused on the postoperative rotational alignment of the entire innominate bone and examined its relationship with acetabular anteversion using three-dimensional computed tomography. By characterizing these morphological parameters, we aimed to clarify whether Pemberton acetabuloplasty is associated with structural changes extending beyond localized acetabular reshaping.

In the present study, Pemberton acetabuloplasty, which utilizes the triradiate cartilage as a hinge, was associated with increased external rotation of the upper pelvis (iliac wing) on the operated side when the contralateral hemipelvis was used as an internal control in unilateral DDH cases. Although this side-to-side comparison reduces inter-individual variability, the contralateral hemipelvis in patients with DDH cannot be assumed to be morphologically normal, as subtle dysplastic or rotational characteristics may be present. Previous studies have reported that the dysplastic hemipelvis in unilateral DDH exhibits relative internal rotational characteristics preoperatively [8–11]. In a side-to-side analysis of unilateral DDH conducted by Jia et al., who first described the three-dimensional CT–based method applied in the present investigation, both upper and lower rotational angles were greater on the dislocated side than on the contralateral side, consistent with relative internal rotation of the affected hemipelvis; however, statistical significance was observed only in the Tönnis grade III subgroup [20]. Within this context, the postoperative external rotation identified in the present study reflects a rotational pattern opposite to the previously described preoperative internal alignment. Accordingly, when interpreted in light of the baseline asymmetry reported in the literature, the magnitude of postoperative external rotation observed in our analysis may appear accentuated in side-to-side comparisons.

Given the excessive external rotation of the upper pelvis (iliac wing) observed following unilateral Pemberton acetabuloplasty, we further evaluated whether a comparable pattern was present in patients who underwent bilateral procedures. No significant differences in pelvic rotational parameters were identified between unilateral and bilateral groups. These findings indicate that bilateral Pemberton acetabuloplasty may be associated with comparable degrees of iliac wing external rotation on both sides, without radiographic evidence of pubic symphysis diastasis on postoperative imaging. To our knowledge, dynamic gait assessments following bilateral Pemberton acetabuloplasty have not been systematically reported. Therefore, the clinical and biomechanical significance of the symmetrical iliac wing external rotation observed on imaging remains unclear and requires further investigation.

Pemberton acetabuloplasty is traditionally described as a reshaping osteotomy. Using patient-specific three-dimensional printed models, Caffrey et al. demonstrated that Pemberton acetabuloplasty increased superior-anterior acetabular coverage but at the same time resulted in relative acetabular retroversion [22]. We further examined the association between postoperative innominate rotation and acetabular anteversion following Pemberton acetabuloplasty. Consistent with these observations, our three-dimensional analysis demonstrated an association between lower pelvic rotational alignment and acetabular version following Pemberton acetabuloplasty, whereas the relationship with upper pelvic rotation was comparatively weaker. Given that smaller lower pelvic rotational angle values correspond to greater external rotation of the lower hemipelvis, this pattern suggests a tendency toward reduced acetabular anteversion in hemipelves demonstrating greater external rotation. Acetabular version has been extensively studied in the context of hip morphology. A retroverted acetabulum has been described as a structural feature associated with pincer-type femoroacetabular impingement, and has been reported to correlate with radiographic features of hip osteoarthritis [23]. Akiyama et al. reported radiographic signs of acetabular retroversion in 37.5% of hips treated with Pemberton acetabuloplasty, a significantly higher frequency than in patients treated with a Pavlik harness alone [24]. In contrast, Bellova et al., using magnetic resonance imaging, found that hips treated with Pemberton acetabuloplasty demonstrated acetabular anteversion comparable to that of contralateral healthy hips [18]. In the present cohort of 20 hips treated with unilateral Pemberton acetabuloplasty, the mean postoperative acetabular anteversion was 15.6° ± 5.8°, which was comparable to 16.0° ± 5.1° measured in the contralateral hips of the unilateral DDH group. Although a slight decrease in anteversion was observed, no cases of acetabular retroversion were identified on postoperative imaging.

The innominate bone provides the origin for multiple muscle groups involved in hip motion, including the gluteal, adductor, lateral rotator, and hamstring muscles, as well as the rectus femoris and sartorius. Prior gait analyses of adolescents treated with unilateral Pemberton acetabuloplasty have reported alterations in pelvic kinematics, such as increased anterior pelvic tilt, pelvic hiking on the operated side, and pelvic rotation toward the contralateral side, along with increased hip joint loading compared with healthy controls [16, 17]. However, the structural correlates underlying these observations remain incompletely defined. Several hypotheses have been proposed to account for postoperative gait alterations, including changes in muscle strength following surgical exposure and modification of pelvic skeletal morphology. Bassett et al., for example, reported atrophy of the iliacus and psoas muscles after open reduction for DDH [25]. In addition, pelvic osteotomies modify bony geometry, which may influence the spatial orientation of muscular origins and insertions. In the present study, excessve external rotation of the upper portion of the operated innominate bone was identified following Pemberton acetabuloplasty, extending beyond localized acetabular reshaping. Nevertheless, because this investigation was limited to structural assessment without dynamic or functional evaluation, no conclusions can be drawn regarding any relationship between these morphological findings and previously reported gait characteristics. Further prospective studies incorporating longitudinal imaging and objective functional assessments are warranted to clarify potential associations between global innominate rotational alignment and postoperative pelvic kinematics or joint loading.

This study has several limitations. First, its retrospective design may have limited the completeness of available clinical and radiographic data. Second, postoperative pelvic CT scans are not routinely performed after Pemberton acetabuloplasty. It may exclude generality of the review. Third, the limited sample size precluded meaningful subgroup analyses to determine whether the observed rotational alignment changes represent early postoperative adaptation, progressive remodeling, or longer-term stabilization, or whether they are associated with an increased risk of revision surgery. Fourth, as this was a single-institution study in which procedures were performed by experienced pediatric orthopedic surgeons, subtle variations in surgical technique may have occurred and could have influenced postoperative pelvic morphology. Finally, as this was a morphology-based study without functional assessment such as gait analysis, the clinical and biomechanical implications cannot be directly established.

## Conclusions

In summary, this study evaluated the postoperative rotational alignment of the entire innominate bone following Pemberton acetabuloplasty using three-dimensional imaging. Excessive external rotation of the upper hemipelvis was observed on postoperative assessment, suggesting that Pemberton acetabuloplasty may be associated with structural changes extending beyond localized acetabular reshaping. Further investigation incorporating longitudinal imaging and functional evaluation is warranted to better characterize the clinical significance of these morphological findings.

## Data Availability

All data generated or analyzed during this study are included in this published article. Anonymized raw datasets are available from the corresponding author upon reasonable request, as privacy and ethical considerations preclude public deposition.

## Abbreviations

DDH: Developmental dysplasia of the hip
3D-CT: Three-dimensional computed tomography
2D-CT: Two-dimensional computed tomography
CT: Computed tomography
SD: Standard deviation
BMI: Body mass index
AA: Acetabular anteversion
URA: Upper pelvic rotational angle
LRA: Lower pelvic rotational angle
ICC: Intraclass correlation coefficients

## References

[1] Ganz R, Leunig M, Leunig-Ganz K, Harris WH. The etiology of osteoarthritis of the hip: an integrated mechanical concept. Clin Orthop Relat Res. 2008;466:264–72. 18196405 10.1007/s11999-007-0060-z PMC2505145

[2] Gala L, Clohisy JC, Beaulé PE. Hip dysplasia in the young adult. J Bone Joint Surg Am. 2016;98:63–73. 26738905 10.2106/JBJS.O.00109

[3] Dandachli W, Kannan V, Richards R, Shah Z, Hall-Craggs M, Witt J. Analysis of cover of the femoral head in normal and dysplastic hips: new CT-based technique. J Bone Joint Surg Br. 2008;90:1428–34. 18978260 10.1302/0301-620X.90B11.20073

[4] Chegini S, Beck M, Ferguson SJ. The effects of impingement and dysplasia on stress distributions in the hip joint during sitting and walking: a finite element analysis. J Orthop Res. 2009;27:195–201. 18752280 10.1002/jor.20747

[5] Hipp JA, Sugano N, Millis MB, Murphy SB. Planning acetabular redirection osteotomies based on joint contact pressures. Clin Orthop Relat Res. 1999;364:134–43. 10.1097/00003086-199907000-00018 10416402

[6] Klaue K, Durnin CW, Ganz R. The acetabular rim syndrome. A clinical presentation of dysplasia of the hip. J Bone Joint Surg Br. 1991;73:423–9. 1670443 10.1302/0301-620X.73B3.1670443

[7] Michaeli DA, Murphy SB, Hipp JA. Comparison of predicted and measured contact pressures in normal and dysplastic hips. Med Eng Phys. 1997;19:180–6. 9203153 10.1016/s1350-4533(96)00051-3

[8] Fujii M, Nakashima Y, Sato T, Akiyama M, Iwamoto Y. Pelvic deformity influences acetabular version and coverage in hip dysplasia. Clin Orthop Relat Res. 2011;469:1735–42. 21203874 10.1007/s11999-010-1746-1 PMC3094603

[9] Sako N, Kaku N, Kitahara Y, Kubota Y, Tagomori H, Tsumura H. Three-dimensional evaluation of innominate bone rotation in female patients with developmental dysplasia of the hip. Clin Orthop Surg. 2022;14:196–204. 35685970 10.4055/cios21032 PMC9152894

[10] Sako N, Kaku N, Tagomori H, Tsumura H. Is the iliac wing curved inward in patients with developmental dysplasia of the hip? Clin Orthop Surg. 2021;13:461–7. 34868494 10.4055/cios20230 PMC8609215

[11] Tannast M, Pfannebecker P, Schwab JM, Albers CE, Siebenrock KA, Büchler L. Pelvic morphology differs in rotation and obliquity between developmental dysplasia of the hip and retroversion. Clin Orthop Relat Res. 2012;470:3297–305. 22798136 10.1007/s11999-012-2473-6 PMC3492631

[12] Venkatadass K, Durga Prasad V, Al Ahmadi NMM, Rajasekaran S. Pelvic osteotomies in hip dysplasia: why, when and how? EFORT Open Rev. 2022;7:153–63. 35192506 10.1530/EOR-21-0066 PMC8897565

[13] Kothari A, Grammatopoulos G, Hopewell S, Theologis T. How does bony surgery affect results of anterior open reduction in walking-age children with developmental hip dysplasia? Clin Orthop Relat Res. 2016;474:1199–208. 26487045 10.1007/s11999-015-4598-x PMC4814424

[14] Huang S-C, Wang T-M, Wu K-W, Fang C-F, Kuo KN. Pemberton osteotomy for acetabular dysplasia. JBJS Essent Surg Tech. 2011;1:e2. 33738127 10.2106/JBJS.ST.K.00003 PMC7821973

[15] Wang C-W, Wu K-W, Wang T-M, Huang S-C, Kuo KN. Comparison of acetabular anterior coverage after Salter osteotomy and Pemberton acetabuloplasty: a long-term followup. Clin Orthop Relat Res. 2014;472:1001–9. 24096458 10.1007/s11999-013-3319-6 PMC3916632

[16] Chang C-F, Wang T-M, Wang J-H, Huang S-C, Lu T-W. Adolescents after Pemberton’s osteotomy for developmental dysplasia of the hip displayed greater joint loading than healthy controls in affected and unaffected limbs during gait. J Orthop Res. 2011;29:1034–41. 21308759 10.1002/jor.21377

[17] Chang C-F, Wang T-M, Wang J-H, Huang S-C, Lu T-W. Residual gait deviations in adolescents treated during infancy for unilateral developmental dysplasia of the hip using Pemberton’s osteotomy. Gait Posture. 2012;35:561–6. 22425193 10.1016/j.gaitpost.2011.11.024

[18] Bellova P, Blum S, Hartmann A, Thielemann F, Günther K-P, Goronzy J. MRI-based assessment of acetabular version and coverage after previous Pemberton osteotomy in skeletally mature patients. J Child Orthop. 2021;15:223–31. 34211598 10.1302/1863-2548.15.210010 PMC8223088

[19] Li LY, Zhang LJ, Zhao Q, Wang EB. Measurement of acetabular anteversion in developmental dysplasia of the hip in children by two- and three-dimensional computed tomography. J Int Med Res. 2009;37:567–75. 19383253 10.1177/147323000903700234

[20] Jia J, Zhang L, Zhao Q, Li L, Liu X. Does medial rotational deformity of the whole pelvis universally exist in unilateral DDH? Arch Orthop Trauma Surg. 2011;131:1383–8. 21617933 10.1007/s00402-011-1326-1

[21] Koo TK, Li MY. A guideline of selecting and reporting intraclass correlation coefficients for reliability research. J Chiropr Med. 2016;15:155–63. 27330520 10.1016/j.jcm.2016.02.012 PMC4913118

[22] Caffrey JP, Jeffords ME, Farnsworth CL, Bomar JD, Upasani VV. Comparison of 3 pediatric pelvic osteotomies for acetabular dysplasia using patient-specific 3D-printed models. J Pediatr Orthop. 2019;39:e159–64. 30300278 10.1097/BPO.0000000000001271

[23] Kim WY, Hutchinson CE, Andrew JG, Allen PD. The relationship between acetabular retroversion and osteoarthritis of the hip. J Bone Joint Surg Br. 2006;88:727–9. 16720763 10.1302/0301-620X.88B6.17430

[24] Akiyama M, Nakashima Y, Oishi M, Sato T, Hirata M, Hara D, et al. Risk factors for acetabular retroversion in developmental dysplasia of the hip: does the Pemberton osteotomy contribute? J Orthop Sci. 2014;19:90–6. 24091985 10.1007/s00776-013-0473-3

[25] Bassett GS, Engsberg JR, McAlister WH, Gordon JE, Schoenecker PL. Fate of the psoas muscle after open reduction for developmental dislocation of the hip (DDH). J Pediatr Orthop. 1999;19:425–32. 10412988 10.1097/00004694-199907000-00002

